# Synthesis, characterization, and sensitivity tests of La_0.8_Ba_0.1_Bi_0.1_FeO_3_ nanoparticles towards a few parts-per-billion of acetone gas

**DOI:** 10.1016/j.heliyon.2024.e26778

**Published:** 2024-02-22

**Authors:** E.M. Benali, L. Saher, A. Benali, M. Bejar, E. Dhahri, Jiangtao Wu, Lin Peng, P.M. Gordo, J. Pina, B.F.O. Costa

**Affiliations:** a Laboratoire de Physique Appliquée, Faculté des Sciences, Université de Sfax, B.P. 1171, 3000, Sfax, Tunisia; b University of Coimbra, CFisUC, Physics Department, Rua Larga, P-3004-516, Coimbra, Portugal; c I3N and Physics Department, University of Aveiro, 3810-193, Aveiro, Portugal; d College of Chemistry and Materials Science, Sichuan Normal University, Chengdu, 610068, China; e Centre de Recherche Scientifique et Technique en Analyses Physico-Chimiques, CRAPC, BP384, Bou-Ismail, 42004, Tipaza, Algeria; f Faculté des Sciences de Monastir, Université de Monastir, Avenue de l’Environnement, 5019, Monastir, Tunisia; g University of Coimbra, CQC-IMS, Department of Chemistry, Rua Larga, 3004-535, Coimbra, Portugal

**Keywords:** Auto-combustion route, FTIR, Bandgap, Positron annihilation spectroscopy, Gas sensors, Acetone, Response/Recovery time

## Abstract

In the present paper, the **La**_**0.8**_**Ba**_**0.1**_**Bi**_**0.1**_**FeO**_**3**_ powders were synthesized via the auto-combustion method. The optical, the positron annihilation spectroscopy and the gas sensing properties of our sample were investigated simultaneously. FTIR spectrum revealed the antisymmetric deformation vibrations of the Fe–O and Fe–O–Fe bonds inside the octahedron FeO_6_. The optical bandgap (E_gap_) of the **La**_**0.8**_**Ba**_**0.1**_**Bi**_**0.1**_**FeO**_**3**_ compound was found to be equal to 2.23 eV. We confirmed by the positron annihilation studies, the existence of open volume defects and vacancy sized defects, at the grain/interfaces between vacancy clusters and grains at the interfaces intersection (triple-lines). Notably, the **La**_**0.8**_**Ba**_**0.1**_**Bi**_**0.1**_**FeO**_**3**_ perovskite exhibits an excellent response toward acetone gas, with ultra-fast response and recovery times to some parts-per-billion (ppb) of this tested gas.

## 1 Introduction

Over the past few years, there has been a soaring rate of environmental pollution. Therefore, the manufacturing of new and efficacious gas sensors towards harmful and deadly gases has become a hot subject in scientific research for the human, animals, and the whole-world safety as well [[1], [2], [3], [4]]. Currently, the semiconductor metal-oxide nanomaterials have been strategically selected for the manufacturing of gas sensors due to their small sizes, cheap cost, and quick recovery/response times towards harmful gases [[5], [6], [7], [8], [9], [10]]. The principal work of gas sensors p-type like semiconductors (such as CuO [11] and CaFe_2_O_4_ [12] or NiO [13]) is the change of resistance on the surface of the sensor when it is exposed to gas. Lanthanum ferrite material, with the general formula LaFeO_3_, is a well-known perovskite oxide material that has shown remarkable physico-chemical properties. It has also been the subject of technological applications, as in gas sensors [[14], [15], [16], [17], [18]]. It is important to note that selectivity, stability, optimal temperature, and sensitivity are the main characteristics and key factors of a practical gas sensor [19].

Several research studies have been conducted to further improve the sensitivity and gas sensing working temperature of the LaFeO_3_-based sensor as p-type semiconductor. For example, a research work reported by J. Qin showed that by ‘’Mg’’ doping, LaFeO_3_ increases the response towards methanol gas [20]. Besides, L. Li et al. revealed that the ‘’Ba’’ doping of LaFeO_3_ enhances the response of this sensor towards CO_2_ toxic gas [21]. Moreover, it was reported that the Ca^2+^ ion insertion in the A-site of LaFeO_3_ material induced a great enhancement of sensitivity to CO gas [22].

It is trusty to note that porous Pb-doped LaFeO_3_ compound synthesized by A. Benali et al. showed better ethanol sensing as well [23]. Indeed, we have recently studied the gas sensing properties of the La_1-2x_Ba_x_Bi_x_FeO_3_ (0.0 < x < 0.2) nanoparticles towards H_2_S and ethanol gases. We also concluded that the La_0.8_Ba_0.1_Bi_0.1_FeO_3_ (x = 0.1) sample, p-type semiconductor material, exhibited the highest responses towards all tested gases with fast response and recovery time values [24]. Moreover, the nanosize criteria of particles in the single phase La_0.8_Ba_0.1_Bi_0.1_FeO_3_ compound was confirmed from the XRD and TEM analyses [24]. It is important to mention that acetone, which is commonly used in the industry and research laboratories has cleaning solvent, and can easily evaporate at ambient temperature, thus causing a massive impact on human health (causing fatigue, narcosis and nerve system damage) as well as environment. Hence, the control of this gas concentrations in the environment is highly required to avoid catastrophic consequences.

Positron Annihilation Spectroscopy (PAS) is considered as one of the most effective characterization tools to study the vacancy-type and material structural defects [25,26]. Indeed, the positron reveals distinct annihilation scenarios within solids, occurring either in delocalized states within defect-free lattices or when trapped in atomic-sized open volume defects. The unique characteristics of PAS enable the extraction of valuable information, as the two emitted gamma rays during electron-positron pair annihilation carry details about electron density and momentum at the annihilation sites. The positron lifetime serves as a distinctive marker for the annihilation site, facilitating the concentration of the defect. Furthermore, the intensity of the lifetime component provides insights into the concentration of the defects within the material.

## 2 Experimental details

### 2.1 Synthesis and gas sensing measurements of La_0.8_Ba_0.1_Bi_0.1_FeO_3_ nanoparticles

The present sample was synthesized using the auto-combustion route. The synthesis details and steps were previously studied [27], where appropriate amounts of Iron nitrate Fe(NO_3_)_3_.9H_2_O, Lanthanum nitrate La(NO_3_)_3_.6H_2_O, Barium nitrate Ba(NO_3_)_2_ and Bismuth nitrate Bi(NO_3_)_3_.5H_2_O were used to obtain the desired La_0.8_Ba_0.1_Bi_0.1_FeO_3_ nanoparticles as shown in the following equation:

1 $$\left(0.8\times La{\left(NO_{3}\right)}_{3}.6H_{2}O\right)+\left(0.1\times Bi{\left(NO_{3}\right)}_{3}.9H_{2}O\right)+\left(0.1\times Ba{\left(NO_{3}\right)}_{2}.4H_{2}O\right)+\left(Fe{\left(NO_{3}\right)}_{3}.9H_{2}O\right)+3.33\left(C_{2}H_{5}NO_{2}\right)\to {La}_{0.8}{Ba}_{0.1}{Bi}_{0.1}FeO_{3}+6.66\left(CO_{2}\right)+{23.425(H}_{2}O)+{4.585\;(N}_{2})$$

**Fig. 1:**
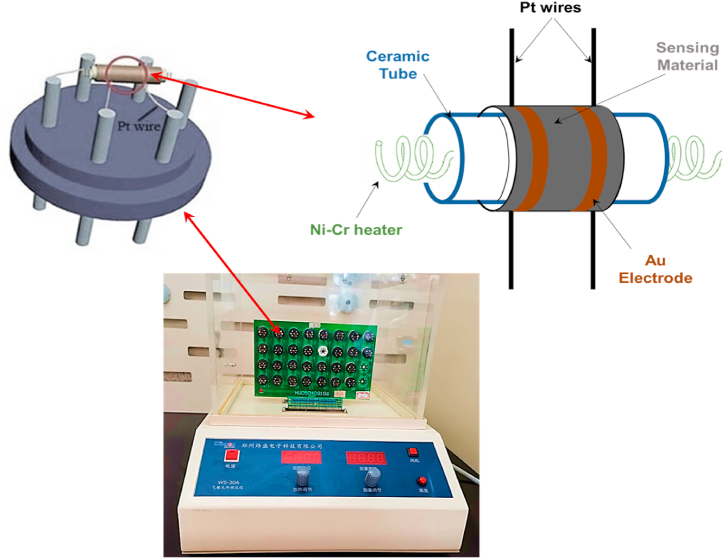
Schematic diagram of the gas-sensing measurement system.

The gas-sensing S-30A system (Zhengzhou Weisen Electronics Technology, P.R. China) was used to carry out the sensitivity measurements towards different gases (Fig. 1). The detailed measurement steps were described in a previous research work [24], exactly as reported by J. Wu et al. [28]. The gas sensing response is defined as S = R_air_/R_gas_ for the n-type semiconductor materials. While, this response is given for the p-type semiconductor materials by the following expression as S = R_gas_/R_air_ (which was used for this work), where R_air_ is the resistance in air and R_gas_ the resistance in the presence of tasted gas [29]**.**

### 2.2 FTIR and U-visible measurements

The infrared spectrum of the selected samples was measured with an Alpha Bruker FTIR spectrometer in the 3750 to 350 cm^−1^ wavenumber range.

The solid-state absorption spectra of the sample were recorded by collecting the total reflectance using a Cary 5000 UV–Vis–NIR spectrophotometer equipped with an integrating sphere (200–2500 nm range). Concerning the background correction. It was performed by collecting the baseline with 100 % and 0 % reflectance (using a Polytetrafluoroethylene, PTFE, reference sample and the blocked beam, respectively) prior to the determination of the spectra of the solid samples. As for the conversion to absorption, it was performed assuming the Kubelka-Munk function, F(R) [30].

### 2.3 Positron spectroscopy

The Positron Annihilation Lifetime Spectroscopy (PALS) measurements were performed using the ^22^Na radioisotope. The procedure involved measuring the time interval between the detection of the 1.28 MeV γ-ray accompanying emission from the ^22^Na radioisotope and the subsequent detection of the 0.511 MeV γ-ray accompanying the positron annihilation. This process facilitated the construction of the lifetime spectra. The PALS setup incorporated a fast-fast coincidence circuit based on Pilot-U scintillators and XP2020 photomultipliers, offering a time resolution of this system of approximately 260 ps (fullwidth at half maximum). The ^22^Na positron source, with an activity of ∼5 μCi was enclosed between two Kapton foils and sandwiched between two identical samples. The measurements were performed at room temperature, yielding several spectra with a total of ∼2 × 10^6^ integral counts per spectrum. The spectra analysis was realized using the LT (version 9) software [31]**.** The source contribution, representing the fraction of positrons annihilating within the source and covering Kapton foils, was determined from the lifetime spectra of annealed pure crystalline silicon (lifetime of 218 ps). Subsequently, the source contribution was recalculated for our samples according to the method described in Ref. [32]**.**

## 3 Results and discussions

### 3.1 Structural properties

**Fig. 2:**
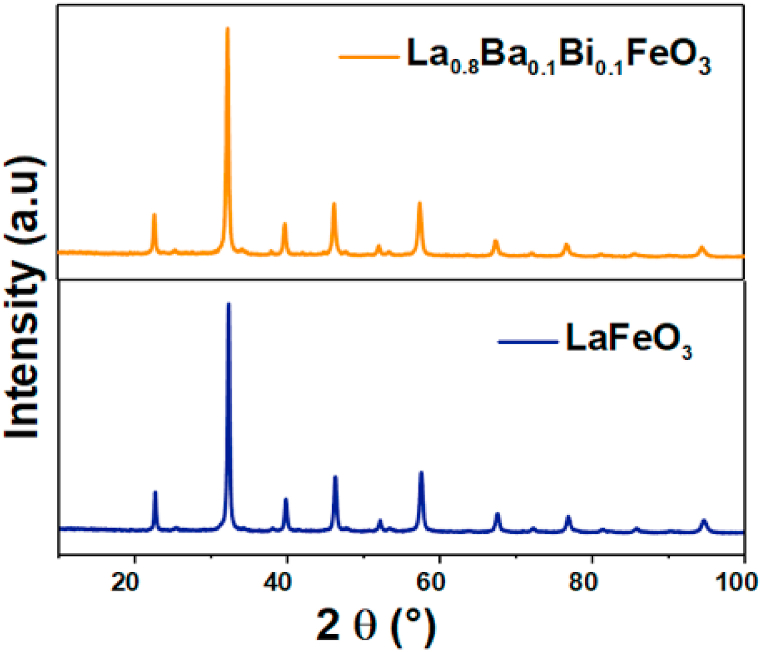
XRD patterns of LaFeO_3_ and La_0.8_Bi_0.1_Bi_0.1_FeO_3_ particles.

The X-ray diﬀraction (XRD) patterns of the La_0.8_Ba_0.1_Bi_0.1_FeO_3_ nanoparticles and the pure LaFeO_3_ are presented in Fig. 2. As previously interpreted [24], our prepared sample presented the same peaks diffraction of the undoped LaFeO_3_.Moreover, this study revealed a well-crystalline orthorhombic structure with *Pnma* space group for this sample, without any secondary phases. The average crystallite size was calculated using the Williamson Hall method [24]**.** The La_0.8_Ba_0.1_Bi_0.1_FeO_3_ compound exhibited the lowest crystallite size.

### 3.2 Optical properties

**Fig. 3:**
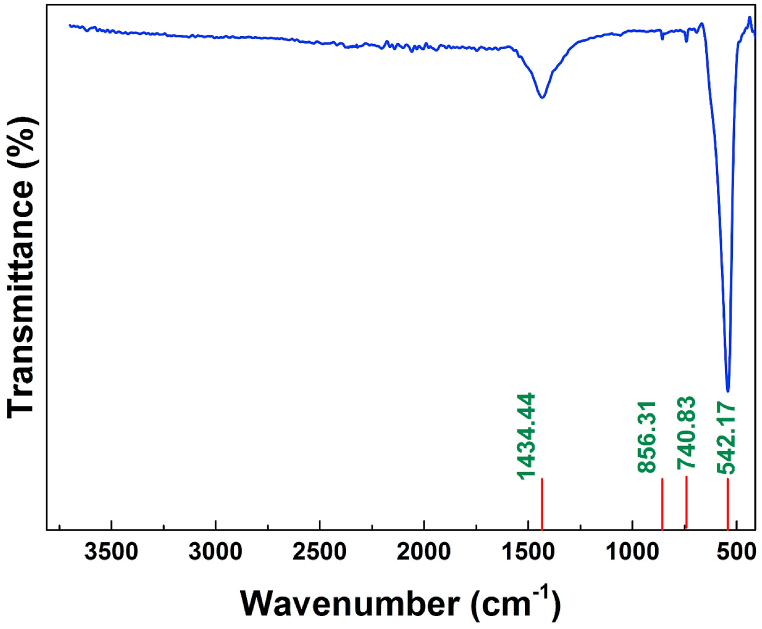
FTIR spectrum of the La_0.8_Ba_0.1_Bi_0.1_FeO_3_ compound.

a) FTIR analysis:

The Fourier transformed infrared (FTIR) spectra corresponding to La_0.8_Ba_0.1_Bi_0.1_FeO_3_ nanoparticles is presented in Fig. 3. Four characteristic peaks are detected in the 300-3700 cm^−1^ wavenumber range. The highest intensive bond observed at 542.14 cm^−1^ represents a distinctive bond of ferrites perovskite materials, specifically associated with the deformation vibrations of Fe–O and the Fe–O–Fe bonds inside the Fe–O_6_ octahedron present in the examined compound [33]. Moreover, the absorption bands that appeared at around 740 cm^−1^ and 856 cm^−1^ are attributed to the bending vibration of the La–O bond and vibrations associated with the ${CO}_{3}^{2-}$ group, respectively. This correlation aligns with prior findings in lanthanum-ferrite systems, confirming the characteristic nature of these vibrational modes [[34], [35], [36]]

In the BiFeO_3_ compound, while the bond at around 540 cm^−1^ is assigned to the vibrational mode of Bi–O bond [37], the one at 1434.44 cm^−1^ is likely to be attributed to the bending modes of the O–H bond [38]. Notably, there is a significant absence of bonds at higher wave numbers especially that at 3600 cm^−1^. This absence contrasts with the findings in LaFeO_3_, produced by the co-precipitation method, where the bond at 3600 cm^−1^ was linked to the La–O bond within the LaFeO_3_ compound made by the co-precipitation method and was attributed to the La–O bond in La–O of the detected secondary phase La_2_O_3_ [38]. The lack of such bonds confirms well the pure phase formation which was previously deduced from structural studies.

**Fig. 4:**
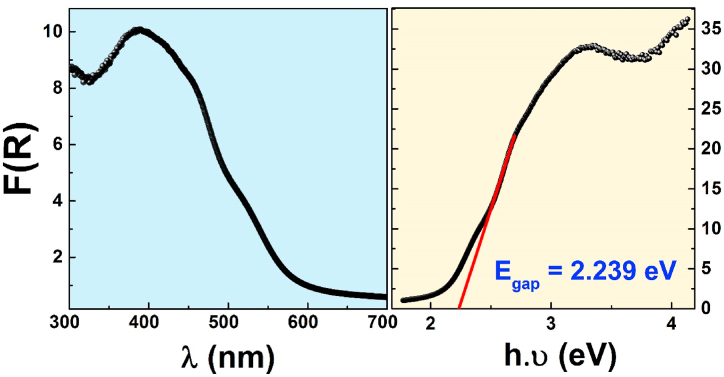
Kubelka-Munk diffuse reflectance UV–Vis spectra and resulting band gaps from Tauc plots of the studied La_0.8_Ba_0.1_Bi_0.1_FeO_3_ sample.

b) UV–vis analysis:

We have employed the UV–Vis diffuse reflectance spectroscopy to investigate the optical properties of the La_0.8_Ba_0.1_Bi_0.1_FeO_3_ nanosized material. The Kubelka-Munk diffuse reflectance UV–Vis (F(R)) spectrum is plotted in Fig. 4. The absorption data were used to calculate the direct band gap energy (E_gap_) by plotting the Tauc plot according to the Kubelka-Munk formalism [39]:

2 $$\alpha =B{\left(h.\upsilon -E_{gap}\right)}^{n}/h.\upsilon$$

with α the absorption-coefficient, ν the irradiation frequency, E_gap_ the band gap, B being a constant (usually 1 for semiconductors), *h* the Planck constant, and n is equal either to 1/2 (for semiconductors with direct transition) or to 2 (indirect transition). It has been reported that the pure LaFeO_3_ semiconductor material exhibits a direct transition with n = 1/2 [40]. Accordingly, Eq. (2) becomes:

3 $${\left(\alpha .h.\upsilon \right)}^{2}=h.\upsilon -E_{gap}$$

The right part of Fig. 4 shows the Tauc plot as a function of (h.ν), revealing that a linear behavior was observed between 2.5 and 3.5 eV, where its linear adjustment gives the band gap energy value, which corresponds to the absorption equal to zero. The deduced E_gap_ value for the studied compound is established at 2.239 eV, closely aligning with values documented in the literature for pure and doped Lanthanum Iron perovskites [[41], [42], [43]]. Finally, this low bandgap value indicates a high potential for practical applications in photocatalytic processes [44].

### 3.3 Positron spectroscopy

**Fig. 5:**
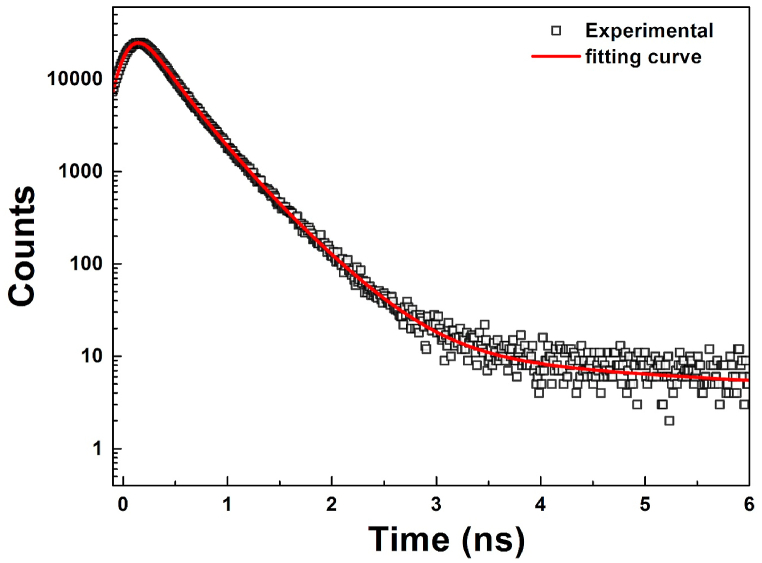
PALS spectrum for La_0.8_Ba_0.1_Bi_0.1_FeO_3_ sample. Experimental data (black square) and fitting curve (red line) for the 3 lifetime components and respective intensity as referred in Table 1. (For interpretation of the references to colour in this figure legend, the reader is referred to the Web version of this article.)

The lifetime spectrum of our sample is presented in Fig. 5. The source contribution was subtracted, and the analysis of the spectrum was realized decompounding it into tree positron lifetime components, $\tau_{i}$, each of which with a certain intensity, $I_{i}$,.

Table 1 summarizes the positron lifetimes and intensities observed in our sample.

**Table 1:** *Positron lifetimes components,*$\tau_{i}$*, and intensities,*$I_{i}$*(%), measured in La*_*0.8*_*Ba*_*0.1*_*Bi*_*0.1*_*FeO*_*3*_*samples. Standard deviations are given in parentheses in units of the last significant digit. It was assumed in the source contribution the contribution of p-Ps with a fix component of 0.125 ns and relative intensity of*$I_{3}$*/3.*

	$\tau_{1}$ (ns)	$I_{1}$ (%)	$\tau_{2}$ (ns)	$I_{2}$ (%)	$\tau_{3}$ (ns)	$I_{3}$ (%)
**La**_**0.8**_**Ba**_**0.1**_**Bi**_**0.1**_**FeO**_**3**_	0.230 (2)	69 (3)	0.410 (5)	30.7 (9)	3.1	0.3 (1)

The observed positron lifetimes components can be divided into 2 groups: the lifetimes group, with $\tau_{1}$ and $\tau_{2}$ components, with lifetime values below 1 ns, and a long lifetime component, $\tau_{3}$, with a value of 3.1 ns. The relative intensity of $\tau_{3}$ component, $I_{3}$, is less than 1% and much smaller than the other 2 components.

The lifetime component $\tau_{3}$ with a value of 3.1 ns can be associated with the annihilation of ortho-positronium (o-Ps) states. The o-Ps atom is a metastable bound state of a positron and an electron with parallel spins, resulting in a triplet configuration. While the theoretical lifetime of o-Ps in vacuum is 142 ns, in the presence of matter, it undergoes a ‘pick-off’ process where the positron is captured by an electron of opposite spin from the surrounding material. This 'pick-off' process effectively reduces the o-Ps lifetime to a few nanoseconds. Necessarily there is a $I_{3}$/3 number of para-positronium (p-Ps) atoms (the spin singlet counterparts with an intrinsic lifetime of 0.125 ns in vacuum). The formation of positronium requires free volumes in the structure of the sample. The $\tau_{3}$ lifetime component arises from the thermalized positrons diffusing to the nanocrystallites surfaces and annihilating as positronium atoms.

From Table 1, it can be concluded that almost all positrons implanted in the perovskite sample annihilate preferentially in two different places, each of which is associated with positron lifetime components $\tau_{1}$ and $\tau_{2}$.

The values observed for $\tau_{1}$ and $\tau_{2}$ (230 ps and 410 ps, respectively) are significantly higher as compared to the typical lifetime of the bulk defect-free material, $\tau_{bulk}$, for the annihilation of delocalized positron in a free defect material.

The observed lifetime values of $\tau_{1}$ and $\tau_{2}$ are consistent with the annihilation of positrons from trapped places associated with open-volume defects. Given the substantial relative values of both lifetime components, it can be assumed that all the positrons are confined within these defects, indicating a saturation trapping regime before the annihilation. The most likely candidates for these defects are vacancies, whose sites must have a negative charge, rendering them potent trapping centers for positrons. This phenomenon elucidates the saturation trapping regime of positrons and, consequently, a high concentration of these defects.

In fact, following positron thermalization within the solid, the positron's thermal diffusion length extends to approximately 50–100 nm [45]. Given that the size of the crystallites in our sample is 31 nm, which is smaller than the positron diffusion length, positrons, upon thermalization tend to become trapped and undergo annihilation within defects predominantly located inside and/or at the grain boundaries of the crystallites. Specifically, the lifetime component $\tau_{1}$ (230 ps) is probably associated with a cation (La) vacancy defect inside the grain and/or along the grain boundaries of the nanocrystals.

The high value of the intensity component $\tau_{1}$ (around 70 %) shows that positrons predominantly annihilate from a trapped state at these defects, suggesting a high concentration of such defects. The vacancies may exist either within the crystallites or at the grain boundary. While the lifetime components of the two vacancy annihilation locations are expected to be different, their values are likely very closely spaced. Therefore, the resolution of the PALS system may not be able to distinguish between both values. It is expected that the component emanating from annihilation at grain boundary vacancy has a higher value [46].

The other component with lifetime $\tau_{2}$ (410 ps) is consistent with the positron annihilation occurring in a larger open-volume defect compared to vacancies. The most probable candidate for this defect is the clusters of few monovacancies. Indeed, in materials with nanocrystalline structure, the grain boundary is known to constitute an important volume fraction, characterized by a chaotic structure with a high concentration of open-volume defects [47]. Due to the small grain size of the nanocrystallites in this sample (smaller than the thermal positron diffusion length), positrons can reach the grain boundaries and become trapped at the defects located at the grain intersections (triple-lines). Similar results have been observed in other nanocrystalline perovskite materials [48,49]

The annihilation of positrons trapped in cationic defects of vacancy cluster type predominantly occurs with 2*p* electrons of negative oxygen ions in the vicinity of the cation vacancy. These charged vacancy cluster defects can play a crucial role in the application of the material, particularly in serving as a gas sensor.

### 3.4 Acetone sensing properties

**Fig. 6:**
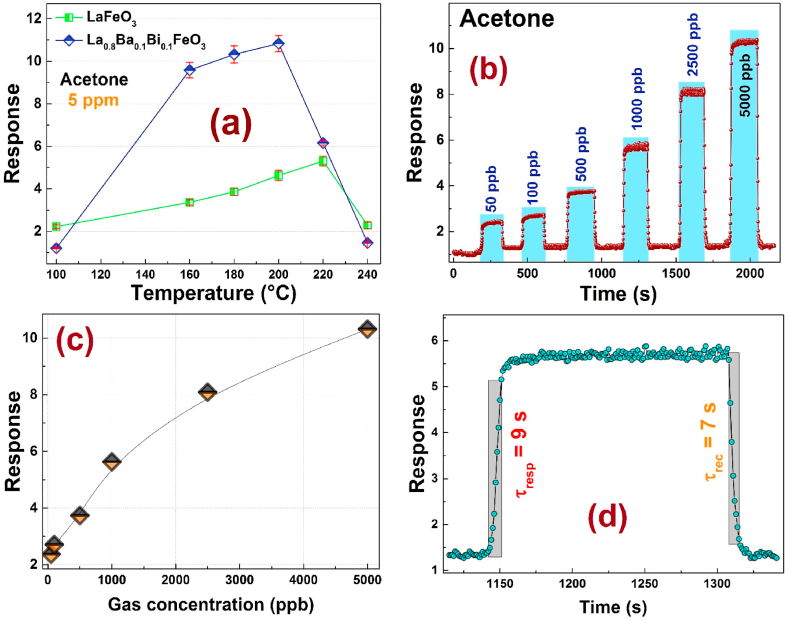
**(a)** Response *vs*. Temperature of the LaFeO_3_ and La_0.8_Ba_0.1_Bi_0.1_FeO_3_ based sensors towards acetone gas; **(b)** Transient response of the La_0.8_Ba_0.1_Bi_0.1_FeO_3_ based sensor exposed to different concentrations of acetone gas at an operating temperature of 200 °C; **(c)** Response vs. acetone gas concentrations; **(d)** Response (τ_resp_) and recovery ((τ_rec_) times of the La_0.8_Ba_0.1_Bi_0.1_FeO_3_ gas sensor to 1000 ppb of acetone gas.

According to the literature, an undoped LaFeO_3_ ceramic-based gas sensor has been found to exhibit a good sensitivity to the acetone gas [[50], [51], [52]]. In this context, the responses of the undoped LaFeO_3_ and the La_0.8_Ba_0.1_Bi_0.1_FeO_3_ compounds towards 5 ppm of acetone vapor were measured across a temperature range of 80–240 °C as presented in Fig. 6 (a). As we can see, the sensor element based on La_0.8_Ba_0.1_Bi_0.1_FeO_3_ compound exhibited an enhanced response to acetone gas as compared with LaFeO_3_.

The results indicate a gradual increase in sensitivity to acetone gas with the elevation of the operating temperature, reaching a maximum value at a selected temperature, followed by a subsequent decline. This observation suggests that our sensor exhibited effective sensing behavior towards acetone vapor.

This volcano trend can be explained by the fact that, at low temperatures, the activation energy of gas molecules is not sufficient enough to overcome the barrier, resulting in a limited reaction with the absorbed oxygen species on the compound surface, and a consequently low response. As the operating temperature rises, the response increases due to the high reaction between gas molecules and absorbed oxygen. However, at a very high temperature, the strong absorption of gas molecules leads to reduced utilization of the sensing material, resulting in a decline in the gas response [53,54].

The La_0.8_Ba_0.1_Bi_0.1_FeO_3_ nanoparticles show a high sensing response to 5 ppm of C_3_H_6_O gas at 200 °C. As compared to the literature results of metal oxide responses, the La_0.8_Ba_0.1_Bi_0.1_FeO_3_ sensor has been proven to exhibit lower operating temperature [55,56].

On the other hand, the transient responses characteristics of the La_0.8_Ba_0.1_Bi_0.1_FeO_3_ sensor towards various acetone-gas concentrations (50, 100, 500, 1000, 2500, and 5000 ppb) at this optimum operating temperature (200 °C) are presented in Fig. 6 (b). Since the response is given by the Rgas/Rair, the sensor is p-type if his response is higher than 1, and the dynamic response curves are deduced from the resistance curves [57].

The response of the La_0.8_Ba_0.1_Bi_0.1_FeO_3_ compound exhibits a rapid increase upon the introduction of the tested gas, reaching a peak value, and subsequently decreases rapidly when the gas is removed (returns to the base line).

Fig. 6 (c) presents the response dependence to the acetone gas concentrations for La_0.8_Ba_0.1_Bi_0.1_FeO_3_ compound. One can easily notice that the increase in tested gas concentration induces an increase in the sensitivity response of the La_0.8_Ba_0.1_Bi_0.1_FeO_3_ compound. This behavior emanates essentially from the increase of the absorbed C_3_H_6_O molecules, which supply more electrons to the surface of the sensor and lead to an increase in the resistance [58]. Table 2 displays the acetone gas response values of previous studies on acetone-sensing materials, comparing them with the results from the current study [[59], [60], [61], [62]]. The comparison affirms that the compound exhibits substantial efficacy in applications related to monitoring acetone gas.

**Table 2:** Gas-sensing performance of various acetone-sensing materials to acetone gas in the literatures and the present study.

Compound	Response	Acetone concentration (ppm)	Operating temperature (°C)	article
2 wt% Pd doped LaFeO_3_	3	5	200	[59]
LaFeO_3_*(Sol-gel)*	0.55	500	275	[60]
La_0.7_Sr_0.3_FeO_3_*(Sol-gel)*	0.7			
biomorphic porous LaFeO_3_*(Sol-gel)*	12.2	200	240	[61]
LaFeO3 thick films	2.1	0.5	260	[62]
La_0.8_Ba_0.1_Bi_0.1_FeO_3_ (Autocombustion)	10.31	5	200	This work

The response/recovery times are known as key parameters used to illustrate the performance of a gas sensor. The response time is defined as the required time for a sensor material to reach 90 % of the maximum response value, while the recovery time represents is the time taken by the sensor to return to the baseline when the tested gas is removed. Fig. 6 (d) depicts the response & recovery times for the La_0.8_Ba_0.1_Bi_0.1_FeO_3_ sample-based sensor to 1000 ppb of acetone at 200 °C. They are found to be equal to 9 s and 7 s, respectively.

#### 3.4.1 Gas sensing mechanism

The resistance of this gas sensor exhibits a low resistance before being in contact with a reductive gas (Acetone). Upon introducing the gas, the resistance undergoes a sharp increase, and after the reaction with the gas, it decreases back to the initial value, after stopping the gas [63]. This behavior is characteristic of the p-type semi-conductor [64], indicating that the gas sensing mechanism is based on the change of the resistance.

When the prepared gas sensor is exposed to air, the oxygen molecules are adsorbed onto the surface of the sensor. These molecules then undergo a reaction with the electrons captured from the conduction band of the material, leading to their transformation into oxygen ions (Eq. (4) to Eq. (6)), and leaving behind holes in bulk La_0.8_Ba_0.1_Bi_0.1_FeO_3_. The reactions of ionization of the oxygen molecules are illustrated in the following (Eq. (4) to Eq. (6)):

4 $$O_{2\;\left(gas\right)}{↔O}_{2\left(ads\right)}$$

5 $$O_{2\left(ads\right)}+e^{-}\;↔\;O_{2\left(ads\right)}^{-}$$

6 $$O_{2\left(ads\right)}^{-}+e^{-}↔\;{2O}_{\left(ads\right)}^{-}$$

when the double doped LaFeO_3_ gas sensor is exposed to acetone gas (reductive gas [65]), a reaction between the molecules of acetone and the ions of oxygen took place, which released the trapped electrons back to La_0.8_Ba_0.1_Bi_0.1_FeO_3_ [66,67], as presented in the following equation (Eq. 7):

7 $${CH}_{3}CO{CH}_{3}+{8O}_{\left(ads\right)}^{-}\;\to \;{3CO}_{2\;}+{3H}_{2}O+{8e}^{-}$$

The produced electrons are recombined with holes (Eq. (8)), which induces a decrease of the holes and increase of the sample resistance [67].

8 $$\dot{h}+\bar{e}\;\to \;null$$

## 4 Conclusion

In the current research work, La_0.8_Ba_0.1_Bi_0.1_FeO_3_ nanosized compound with perovskite structure was successfully prepared using a facile auto-combustion method. This compound exhibits a low bandgap energy, making it suitable for use in photocatalytic applications. The Positron Annihilation Lifetime measurements have revealed that the La_0.8_Ba_0.1_Bi_0.1_FeO_3_ samples are rich in open-volume defects, particularly, vacancy-sized defects at grain/grain boundaries and clusters of vacancies at interfaces between the grains. Additionally, the La_0.8_Ba_0.1_Bi_0.1_FeO_3_ nanoparticles demonstrate elevated response values towards ppb acetone gas concentrations, characterized by an ultrafast response and recovery times.

## Data availability statement

Data will be made available on request.

## Ethics statement

Review and/or approval by an ethics committee was not needed for this study because animals or humans subjects/cells was not used.

## CRediT authorship contribution statement

**E.M. Benali:** Writing – original draft, Investigation, Conceptualization. **L. Saher:** Writing – original draft, Investigation, Conceptualization. **A. Benali:** Writing – original draft, Investigation, Conceptualization. **M. Bejar:** Writing – review & editing. **E. Dhahri:** Writing – review & editing. **Jiangtao Wu:** Funding acquisition, Formal analysis. **Lin Peng:** Funding acquisition, Formal analysis. **P.M. Gordo:** Writing – original draft, Validation, Formal analysis. **J. Pina:** Writing – review & editing, Validation, Formal analysis. **B.F.O. Costa:** Writing – review & editing, Formal analysis, Conceptualization.

## Declaration of competing interest

The authors declare that they have no known competing financial interests or personal relationships that could have appeared to influence the work reported in this paper.
